# Editorial: Neuroimaging of neuroinflammation in neurological disorders

**DOI:** 10.3389/fneur.2023.1328511

**Published:** 2023-11-10

**Authors:** Md Nasir Uddin, Madalina E. Tivarus, Giovanni Schifitto, David A. Rudko

**Affiliations:** ^1^Department of Neurology, University of Rochester, Rochester, NY, United States; ^2^Department of Biomedical Engineering, University of Rochester, Rochester, NY, United States; ^3^Department of Imaging Sciences, University of Rochester, Rochester, NY, United States; ^4^Department of Neuroscience, University of Rochester, Rochester, NY, United States; ^5^Department of Electrical and Computer Engineering, University of Rochester, Rochester, NY, United States; ^6^McConnell Brain Imaging Centre, Montreal Neurological Institute and Hospital, Montreal, QC, Canada; ^7^Department of Neurology and Neurosurgery, McGill University, Montreal, QC, Canada; ^8^Department of Biomedical Engineering, McGill University, Montreal, QC, Canada

**Keywords:** neuroimaging, neuroinflammation, CNS disorders, MRI, brain

Neuroinflammation is an alteration of central nervous system (CNS) homeostasis in response to pathogens, trauma, stroke, neoplasms, systemic inflammation, and neurodegenerative disorders. A neuroinflammatory response is also standard during human aging. While microglial activation represents the resident immune cell response to injury in the CNS, reactive astrocytes, as well as alteration of the blood brain barrier and neurovascular unit are also tightly connected to the inflammatory response. Further, transmigration of activated monocytes and differentiation of perivascular macrophages also contribute to chronicity of neuroinflammation (1–4).

Neuroinflammation plays a central role in the onset of several neurological disorders, including Multiple Sclerosis (MS), Parkinson's disease, Alzheimer's disease, and HIV infection (1). Its role in the onset of these conditions has yielded an increasing demand for targeted treatments toward neuroinflammation and improved strategies for quantitative imaging and monitoring that are more definitively relevant for clinical diagnoses. Recent advances in neuroimaging provide several non-invasive or minimally invasive methods, many of which incorporate MRI, for more advanced insight into neuroinflammatory processes within the CNS.

The objective of this Research Topic was to emphasize the significance of advanced neuroimaging techniques and measures for quantifying neuroinflammation. The articles contained in this Research Topic also explore clinical and neurocognitive implications of neuroinflammation across a range of neurological conditions. In particular, the primary aims include: (a) Introducing innovative imaging techniques for robust neuroinflammation quantification, (b) Proposing biophysical models through single or multimodality imaging approaches and multimodal integration to enhance our understanding of neuroinflammatory processes, (c) Investigating the connections between imaging markers and neuroinflammatory blood markers or neurocognitive performance, (d) Providing a comprehensive and detailed examination of relevant imaging techniques, along with their capabilities and limitations in assessing neuroinflammation.

The present Research Topic encompasses eight articles, including five original research studies, two case reports, and one methods article. These contributions were provided by a collective of 61 authors, representing four different countries. The studies collectively offer diverse perspectives and ideas that significantly enhance our comprehension of the field.

Liang et al. investigated the diagnostic potential of a unique 3 Tesla, resting-state fMRI-derived metric—the amplitude of low-frequency neural fluctuations (2d-fALFF). Their work specifically integrated relationships between 2d-fALFF and cortical thickness. The authors employed a support vector machine (SVM) to understand the potential of 2d-fALFF for distinguishing active MS, inactive MS, and healthy controls (HC).

Carter et al. explored changes in functional connectivity (FC) between individuals with MS and HC, identifying significant differences in FC for specific brain regions associated with emotion, cognition, vision, and language. This was carried out using a whole-brain region-of-interest (ROI) approach. Their findings underscore the relevance of regional FC analysis for understanding information processing speed impairments in MS.

Arterial stiffness may alter the brain microcirculation and is considered a significant factor associated with a multitude of neurodegenerative diseases, including Alzheimer's and other dementias. Arterial stiffness is also linked to concurrent cognitive impairments. In the study by Laporte et al., correlations were explored between arterial stiffness, characterized by arterial pulse wave velocity between the femoral and carotid arteries, and white matter myelination parameters, measured via MRI. The research involved a cohort of 38 cognitively unimpaired healthy individuals, aged between 22 and 94. The results of the work revealed strong correlations between pulse wave velocity and both aggregate g-ratio and myelin volume fraction across 10 white matter ROIs. They concluded that arterial stiffness is associated with relevant changes in myelination, implying that controlling arterial stiffness could serve as a potential therapeutic target for preserving the health of white matter tissue in cerebral normative aging.

Sun et al. aimed to identify the differences in spinal cord gadolinium enhancement features between Neuromyelitis Optica spectrum disorder (NMOSD) and long-segment Degenerative Cervical Myelopathy (DCM). Both conditions can present similarly on MRI scans. They demonstrate that NMOSD and DCM can be well-differentiated by analyzing the number, length, location and form of the gadolinium enhancements.

Singh et al. investigated longitudinal changes in cerebrovascular reactivity (CVR), cerebral blood flow (CBF), endothelial markers, and glial microparticles (MPs) before and after the initiation of combined antiretroviral therapy (cART). The study encompassed 35 cART-naïve individuals living with HIV (PWH) who were assessed at baseline, 12 weeks, and 2 years following cART initiation. Additionally, 53 seronegative controls underwent assessments at corresponding time points. Discrepancies were detected in both CVR and blood biomarkers between PWH and the control group across all time points. Their overall findings indicate that, while cART treatment initially improves neurovascular function, such benefits wane over time.

Lin et al. presented a unique case of coexisting autoimmune Glial fibrillary acidic protein (GFAP) astrocytopathy and reversible splenial lesion syndrome (RESLES), thus contributing to the growing body of evidence in this field. As well, Lotti et al. revealed the utility of 3D intracranial vessel wall imaging (VWI) for detecting and monitoring CNS inflammation in Susac syndrome (SS).

Lastly, Alsameen et al. introduced the Constrained neurite orientation dispersion and density imaging (C-NODDI) method, addressing an essential aspect of NODDI (5, 6) quantification and its relevance to the aging process. Importantly, the C-NODDI approach does not require additional acquisition time and utilizes standard clinical diffusion MRI protocols. C-NODDI serves as a complementary method to NODDI, particularly for determining neurite density index (NDI) in white matter. Their analysis revealed significant quadratic associations of NDI and age in several white matter regions. In addition, compared to conventional NODDI analysis, C-NODDI-NDI values exhibited a stronger correlation with neurofilament light chain (NfL) concentration levels, with lower NDI values corresponding to higher NfL levels.

In summary, we believe the innovative studies contained in this Research Topic significantly exemplify recent advances in the field of neuroimaging of neuroinflammation. We hope the insights gained from this Research Topic will inspire, inform, and guide researchers in the field.

## Author contributions

MU: Writing—original draft, Writing—review & editing. MT: Writing—review & editing. GS: Writing—review & editing. DR: Writing—review & editing.
